# Bevacizumab Plus FOLFOX-4 Combined With Deep Electro-Hyperthermia as First-line Therapy in Metastatic Colon Cancer: A Pilot Study

**DOI:** 10.3389/fonc.2020.590707

**Published:** 2020-11-03

**Authors:** Girolamo Ranieri, Carmelo Laface, Mariarita Laforgia, Simona De Summa, Mariangela Porcelli, Francesco Macina, Michele Ammendola, Pasquale Molinari, Gianfranco Lauletta, Alessandra Di Palo, Giuseppe Rubini, Cristina Ferrari, Cosmo Damiano Gadaleta

**Affiliations:** ^1^ Interventional and Medical Oncology Unit, IRCCS Istituto Tumori “G. Paolo II”, Bari, Italy; ^2^ Pharmacy Unit, IRCCS Istituto Tumori “G. Paolo II”, Bari, Italy; ^3^ Molecular Diagnostics and Pharmacogenetics Unit, IRCCS-Istituto Tumori “Giovanni Paolo II”, Bari, Italy; ^4^ Department of Health Science, Digestive Surgery Unit, University “Magna Graecia” Medical School, Germaneto, Italy; ^5^ Department of Biomedical Sciences and Human Oncology, Section of Internal Medicine “G. Baccelli”, University of Bari Medical School, Bari, Italy; ^6^ Nuclear Medicine Unit, D.I.M., University of Bari “Aldo Moro”, Bari, Italy

**Keywords:** tumor angiogenesis, bevacizumab, hyperthermia, chemotherapy, metastatic colon cancer

## Abstract

Bevacizumab plus FOLFOX-4 regimen represents the first-line therapy in patients affected by metastatic colorectal cancer (mCRC). Hyperthermia has been considered an effective ancillary treatment for cancer therapy through several anti-tumor mechanisms, sharing with Bevacizumab the inhibition of angiogenesis. Up to now, scientific literature offers very few clinical data on the combination of bevacizumab plus oxaliplatin-based chemotherapy with deep electro-hyperthermia (DEHY) for metastatic colon cancer (mCC) patients. Therefore, we aimed at evaluating the efficacy of this combination based on the possible interaction between the DEHY and bevacizumab anti-tumor mechanisms. We conducted a retrospective analysis on 40 patients affected by mCC treated with the combination of bevacizumab plus FOLFOX-4 (fluorouracil/folinic acid plus oxaliplatin) and DEHY (EHY2000), between January 2017 and May 2020. DEHY treatment was performed weekly, with capacitive electrodes at 80–110 W for 50 min, during and between subsequent bevacizumab administrations, on abdomen for liver or abdominal lymph nodes metastases and thorax for lung metastases. Treatment response assessment was performed according to the Response Evaluation Criteria for Solid Tumors (RECIST). The primary endpoints were disease control rate (DCR) and progression-free survival (PFS). The secondary endpoint was overall survival (OS). DCR, counted as the percentage of patients who had the best response rating [complete response (CR), partial response (PR), or stable disease (SD)], was assessed at 90 days (timepoint-1) and at 180 days (timepoint-2). DCR was 95% and 89.5% at timepoint-1 and timepoint-2, respectively. The median PFS was 12.1 months, whereas the median OS was 21.4 months. No major toxicity related to DEHY was registered; overall, this combination regimen was safe. Our results suggest that the combined treatment of DEHY with bevacizumab plus FOLFOX-4 as first-line therapy in mCC is feasible and effective with a favorable disease control, prolonging PFS of 2.7 months with respect to standard treatment without DEHY for mCC patients. Further studies will be required to prove its merit and explore its potentiality, especially if compared to conventional treatment.

## Introduction

Colorectal cancer (CRC) is the fourth most frequently diagnosed cancer and the second leading cause of cancer-related death in the United States (1). In 2019, approximately 49,000 new cases of CRC were diagnosed in Italy, while about 20,000 people died of CRC during 2016 (2). CRC is the second leading cause of cancer in both sexes in Italy, with a 5-year survival rate of 65% (2). Despite these high numbers, the incidence and mortality of CRCs decreased during the last decades, thanks to cancer prevention, earlier diagnosis through preventing screening and better treatment approaches (1, 2).

Approximately 50 to 60% of patients affected by CRC develop metastases (3–5), and the liver represents the most frequent metastatic site (6), leading to death in most patients (7).

Both oral and intravenous fluoride pyrimidines, irinotecan, oxaliplatin, anti-EGFR, and antiangiogenic monoclonal antibodies, regorafenib, trifluridine/tipiracil (TAS-102) and, in very dated studies, mitomycin C proved to be effective in the treatment of advanced disease (8–17).

The patients’ health conditions drive the choice of the appropriate recommended first-line basic schedules which include intensive therapies, such as FOLFOX, XELOX, FOLFIRI, and FOLFOXIRI (8–14). In addition, biologic agents such as bevacizumab, cetuximab or panitumumab, can be combined to chemotherapy depending on the K-RAS biomarker status of the tumor. Further systemic therapies for patients with progressive disease always depend on the chosen first-line therapy.

Bevacizumab has been the first recombinant humanized murine IgG1 monoclonal antibody blocking the biomolecular activity of all the isoforms of the circulating Vascular Endothelial Growth Factor A (VEGF-A), a natural ligand that plays a pivotal role in tumor angiogenesis, being up-regulated in several human tumors (18–22). In particular, bevacizumab inhibits the VEGF/VEGF receptor signaling pathway, blocking tumor angiogenesis (20, 23) decreasing microvessel density but inducing HIF-1 gene expression (24), which is a fleeting molecular balance that stimulates VEGF activity. Some recent studies have demonstrated that non-responder colorectal cancer patients had high pre-treatment HIF-1 levels (25).

Since 2004 FDA has recognized its revolutionary mechanism of action, approving its clinical use in several tumor diseases, and the first was mCRC.

The activity of bevacizumab as first-line therapy in mCRC was evaluated in several randomized phase II and III studies with significant improvements in clinical outcomes (8–11). In more detail, NO16966 trial is a phase III study comparing XELOX plus bevacizumab or placebo *versus* FOLFOX plus bevacizumab or placebo as first therapy in patients with mCRC (12). Clinical results confirm that bevacizumab plus oxaliplatin-based chemotherapy regimens increase progression-free survival (PFS) of 1.4 months with respect to the same regimens without bevacizumab, while no statistical significance difference in overall survival (OS) was reached (12).

Bevacizumab toxicity is far from the common cytotoxic chemotherapy-associated side effects, such as myelosuppression, alopecia, diarrhea, nausea, and vomiting (26). It is rather associated with proteinuria, hypertension, arterial thromboembolic events, wound healing complications, bleedings, and gastrointestinal perforation (11).

In the cases of liver or lung metastases from CRC, loco-regional therapies often play an additional key role along the cure pathway: surgery is the gold standard treatment for resectable metastases, while tumor ablation is indicated for non-surgery eligible patients or for small metastases that can be treated with adequate margins (27–32). Ablative techniques include radiofrequency ablation, microwave ablation, cryoablation, and irreversible electroporation (2, 32–36).

In contrast, liver-only or liver-dominant metastatic disease not eligible for surgery or ablation can be a candidate for locally arterial directed treatments, such as hepatic arterial infusion chemotherapy, yttrium-90 microsphere radioembolization, and transcatheter arterial chemoembolization (2, 37–47).

Among other physical treatments, hyperthermia (HT) proved to be an effective anti-tumor approach in combination with standard therapies. HT increases the temperature of tumor tissue up to 40–45°C and is applied as an enhancer of the effects both of radiotherapy and, to a lesser degree, of chemotherapy, in the treatment of different tumors, such as breast cancer, cervix carcinoma, head and neck cancer, glioblastoma, melanoma, peritoneal carcinomatosis, hepatocellular carcinoma, and soft tissue sarcoma, with significant improvements in clinical outcomes (48–56). Multiple direct and indirect mechanisms are responsible for the synergistic anti-cancer effect performed by HT. First of all, it has an evident cytotoxic action on cancer cells living in hypoxic, nutrient-deprived, and acid microenvironments (57, 58). Secondly, HT enhances the activity of anti-cancer drugs, by influencing plasmatic membrane protein distribution and transmembrane efflux pumps (59), so that the increased membrane permeability facilitates the uptake of antineoplastic agents within cancer cells (59). Thirdly, the application of a higher temperature in a specific area of the body is responsible for the denaturation of intracellular proteins, the inhibition of repair enzymes implying alteration of DNA repair processes and the expression of heat-shock proteins (HSPs) (60, 61). The activation of HSP-mediated pathways determinates the induction of apoptosis and other cell-death mechanisms (62). Fourthly, HT hinders DNA homologous recombination, preventing the reparation of DNA breaks due to chemotherapy (63). Therefore, the combination of chemotherapy and hyperthermia boosts up DNA damage in the tumoral cells, selectively. There are several *in vitro* studies that have demonstrated the increased cytotoxicity of several chemotherapeutic agents, thanks to thermal exposure, such as platinum, melphalan, fluorouracil, and doxorubicin (64–66). Fifthly, HT induces a local vasodilatation which brings a greater drug dose into the tumor area. Finally, HT inhibits tumor angiogenesis through two different mechanisms. On the one hand, it directly damages endothelial cells because of the absorption of the electric field related thermal energy in the extracellular liquid, with a subsequent temperature gradient between the extra- and intracellular compartments, which threatens and/or destroys cancer cell membranes (67, 68). On the other hand, it is well known that hypoxic tumor microenvironment stimulates the expression of hypoxia-inducible factor-1 (HIF-1), which is the main VEGF inducer, the most powerful angiogenic factor. Moreover, HIF-1 also induces the expression of genes involved in an exceeding metabolism, shifting cells towards glycolysis and reducing oxygen consumption rate (69–71). In contrast, HT favors reoxygenation and down-regulates the expression of HIF-1, both through vasodilatation that enhances tumor perfusion and by decreasing oxygen consumption (70, 72, 73).

Deep electro-hyperthermia (DEHY), also known as oncothermia, is a method of locoregional HT. DEHY works by generating a modulated electric field with a carrier radiofrequency of 13.56 MHz through two active electrodes. Since malignant tissue has higher conductivity than healthy human tissue, the electric field tends to flow predominantly through the malignant tumor tissue. Thanks to the interaction between the electric field and the heat, selection at the cellular level takes place, and the system self-focuses on the tumor. In fact, the electric field tends to move through the pathways with the lowest impedance, *i.e*. through the malignant tissue (71, 74–77).

To the best of our knowledge, scientific literature offers very few data on the efficacy of anti-angiogenetic agents plus chemotherapy combined with HT (78).

Based on promising clinical results, its multiple anti-cancer mechanisms and possible unexplored advantages of its combination with an anti-angiogenic agent, we evaluated the synergic efficacy of DEHY in combination with bevacizumab plus FOLFOX-4 (fluorouracil/folinic acid plus oxaliplatin) as first-line therapy in 40 patients affected by metastatic colon cancer.

## Patients and Methods

### Patient Population

**Figure 1:**
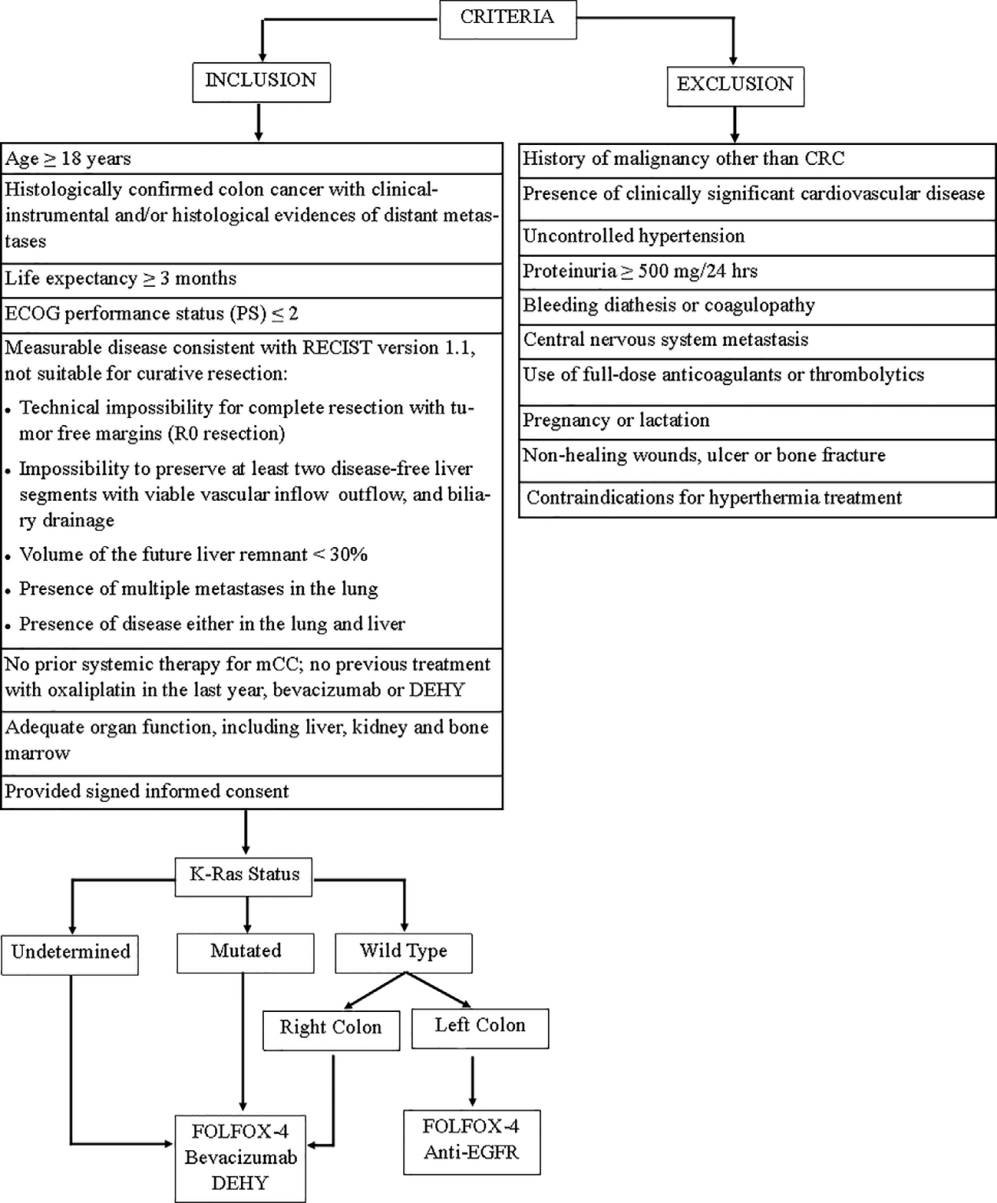
Flow diagram of study inclusion/execution criteria. FOLFOX-4, fluorouracil/folinic acid plus oxaliplatin; DEHY, Deep electro-hyperthermia.

Forty patients with untreated mCC were referred to the “Interventional and Medical Oncology Unit” of the National Cancer Research Centre, Istituto Tumori “Giovanni Paolo II” in Bari (Italy) between January 2017 and May 2020. Patients who met the following criteria were included in this study: (1) patients age ≥18 years with histologically confirmed colon cancer with clinical–instrumental and/or histological evidence of distant metastases; (2) life expectancy ≥3 months; (3) Eastern Cooperative Oncology Group (ECOG) performance status (PS) ≤2; (4) measurable disease consistent with the Response Evaluation Criteria in Solid Tumors (RECIST) version 1.1, not suitable for curative resection based on surgical criteria (**Figure 1**); (5) no prior systemic therapy for mCC; no previous treatment with oxaliplatin in the last year, bevacizumab or DEHY; (6) adequate organ function, including liver, kidney, and bone marrow; (7) provided signed informed consent.

The key exclusion criteria were: (1) history of malignancy other than CRC; (2) the presence of clinically significant cardiovascular disease; (3) uncontrolled hypertension; (4) proteinuria ≥500 mg/24 h; (5) bleeding diathesis or coagulopathy; (6) central nervous system metastasis; (7) use of full-dose anticoagulants or thrombolytics; (8) pregnancy or lactation; (9) non-healing wounds, ulcer, or bone fracture; (10) contraindications for hyperthermia treatment.

Patients with no completed clinic-pathological and survival data were also excluded.

**Figure 1** is a flow diagram of study inclusion/execution criteria.

### Bevacizumab Plus FOLFOX-4 Regimen

All patients received bevacizumab plus FOLFOX-4 regimen as first-line therapy for metastatic disease. FOLFOX-4 schedule includes on day 1, Oxaliplatin intravenous infusion at a dose of 85 mg/m^2^ dissolved in glucose 5% was administered over 120 min contemporary to Leucovorin 200 mg/m^2^ dissolved in glucose 5%. Leucovorin was given also on day 2 before fluoropirimidine. 5-Fluorouracil, as intravenous bolus at the dosage of 400 mg/m^2^, was administered before its intravenous continuous infusion over 22 h at the dosage of 600 mg/m^2^/die on days 1 and 2. As for bevacizumab, intravenous administration at a dose of 5 mg/kg in 100 ml sodium chloride 0.9% was administered before Oxaliplatin over 90 (for the first time) and 60 (for the sequent infusions) min on day 1 and repeated every 14 days.

Anti-emetic prophylaxis was conducted with a serotonin-5HT3-antagonist.

Treatment was continued until disease progression or unacceptable drug-related toxicities (by oxaliplatin above all), considering the shift to bevacizumab alone or plus 5-FU/LV as maintenance therapy.

### Deep Electro-Hyperthermia

DEHY was performed by using the Oncotherm EHY-2000 medical device (Oncotherm GmbH, Traisdorf, Germany). Oncotherm EHY-2000 is made up of three components: a therapy bed with built-in waterbed mattress, a generator unit, and a web box system. The system’s electronics is housed in the generator unit. A mobile computer unit allows viewing and saving the treatment data.

A modulated electric field with a carrier radiofrequency of 13.56 MHz is generated by two active electrodes: the large bolus electrode (30 cm in diameter) positioned at the site where the patient is to be treated and the counter electrode positioned under the mattress of the waterbed. During treatment, the patient lies on the waterbed and becomes part of the electric field *via* the bolus electrode.

DEHY was performed at an output power of 80–110 W generated by the generator unit, obtaining a calculated temperature of 41.5–42°C for 50 min as the whole hyperthermia time, including the 2–3 min of preheating until the therapeutic temperature is reached.

A water bag was used to protect the skin from overheating.

All patients received DEHY treatment weekly, during and between subsequent bevacizumab administrations. The target area of DEHY was the abdomen (n = 36) for liver or abdominal lymph nodes as sites of metastasis and thorax (n = 10) for lung metastasis on the basis of CT imaging guidance. If more than one target area was present, a maximum of two target sites were used alternately, each in one of the two DEHY treatments within a cycle (n = 9). Patients were carefully instructed to report any discomfort during treatment. Moreover, late DEHY-associated adverse events (AEs) were recorded for each patient. DEHY treatment was stopped if an adverse event occurred or by patients will.

### Assessments

A clinical–instrumental evaluation based on general condition, clinical signs, laboratory tests, chest–abdomen–pelvis contrast enhancement computed tomography (ceCT) scan were required before starting the treatment after 90 days (timepoint-1), 180 days (timepoint-2), and then every 3 months to assess tumor response, monitor safety, compliance and determine AEs. Moreover, ^18^F-FDG PET/CT at baseline and at timepoint-2 was performed to all patients. Two different radiologists and nuclear physicians evaluated and checked all ceCT and ^18^F-FDG PET/CT exams independently.

Dimensional tumor measurements were performed on ceCT according to Response Evaluation Criteria for Solid Tumors (RECIST-Version 1.1) (79), and treatment response was indicated as Complete Response (CR), Partial Response (PR), Stable Disease (SD), and Progressive Disease (PD).

AEs were estimated according to Common Terminology Criteria for Adverse Events (CTCAE) version 4.0 and reported in the clinical folder at each cycle of treatment. A decrease of white blood cell counts below 2 × 10^3^ µl, of granulocytes below 0.5 ×10^3^ µl, and of platelets below 100 ×10^3^ µl implied a treatment delay of 1 week or more. Chemotherapy doses were reduced in the following cycle to 75% if nadir of granulocytes was <1.5 × 10^3^ µl, platelets <100 × 10^3^ µl, or any non-hematological toxicity grade 3 occurred.

### Statistical Analysis

The primary endpoints were disease control rate (DCR) and PFS. The secondary endpoint was OS. DCR was considered as the percentage of patients who had the best response rating [complete response (CR), partial response (PR), or stable disease (SD)] and was assessed at 90 days (timepoint-1) and at 180 days (timepoint-2).

PFS was defined as the time from the start of treatment until the date of the first radiological evidence of PD or the date of death derived from any cause, whichever occurred first. OS was specified as the time from the start of treatment until the date of death.

Fisher’s exact test was used to assess the correlation between DCR, PFS, OS, and tumor location (left-sided CRC/right-sided CRC), K-RAS status (wild type/mutation), number of metastatic sites (1–2, ≥3), liver involvement (yes/no), and/or lung involvement (yes/no). R *barplot()* function was used to create barplots. For survival analyses, the Kaplan–Meier method was used to estimate the correlation between PFS, OS rates, and clinic-pathological variables at 95% CI. The log-rank test was used to compare survival curves. The “survival” R package has been used to perform survival analyses. Cox proportional-hazards regression test using the ‘coxph’ function of the R ‘survival’ package has been elaborated. Survival curves have been graphically depicted by “ggplot 2” R package. All statistical analyses were performed using R version 3.6.

## Results

### Patient Characteristics

Forty patients affected by mCC (21 female, 19 male; median age 64.4 years old) treated with bevacizumab plus FOLFOX-4 combined with DEHY between January 2017 and May 2020 in the “Interventional and Medical Oncology” of the National Cancer Research Centre, Istituto Tumori “Giovanni Paolo II” in Bari (Italy) were collected and retrospectively analyzed.

Patients presented an ECOG PS of 0 (n = 25), 1 (n = 11), 2 (n = 4) at the first therapy administration. Among 40 patients, five patients had primitive tumor in site (12.5%), while 35 patients had previously undergone resection of primitive tumor (87.5%); 26 patients (65%) harbored left-sided CC, 27 (67.5%) were KRAS mutated, 32 (80%) had ≤2 metastatic sites with liver as the most common metastatic organ (36 patients, 90%). All patients were not eligible for surgery because of unresectable disease according to abovementioned criteria. Patients’ characteristics are shown in **Table 1**.

**Table 1:** Baseline patient Characteristics.

Characteristics	Enrolled Patients (n = 40)	%
Gender		
Male	19	47.5%
Female	21	52.5%
Median age, years	64,4	Range 45–80
ECOG Performance Status		
0	25	62.5%
1	11	27.5%
2	4	10%
Primitive tumor in site		
Yes	5	12.5%
No	35	87.5%
Primitive tumour side		
Right Colon	13	32.5%
Left Colon	27	67.5%
Biomarker status (K-Ras)		
Wild Type	13	32.5%
Mutated	27	67.5%
No. of metastatic sites		
1–2	32	80%
≥3	8	20%
Major involvement site		
Liver	36	90%
Lung	4	10%

### Efficacy

Clinical–instrumental evaluation at timepoint-1 was assessed in all patients of our study: PR was detected in 12/40 (30%) patients, SD in 26/40 (65%) patients, and PD in 2/40 (5%) patients, with a DCR of 95% (**Table 2**).

**Table 2:** Clinical response assessment according to RECIST at timepoint-1 and timepoint-2.

Clinical Response	Timepoint 1 (*n* = 40)	%	Timepoint 2 (*n* = 38)	%
CR	/	/	2	5.3
PR	12	30	10	26.3
SD	26	65	22	55
PD	2	5	4	10.5
DCR	38	95	34	89.5

38 patients (95%) completed the clinical–instrumental evaluation at timepoint-2: CR was achieved in 2/38 (5.3%) patients, PR in 10/38 (26.3%) patients, SD in 22/40 (55%) patients, and PD in 4/38 (10%) patients, with a DCR of 89.5% (**Table 2**).

DCR decreased of 5.55% from timepoint-1 to timepoint-2 treatment response evaluations.

**Figure 2:**
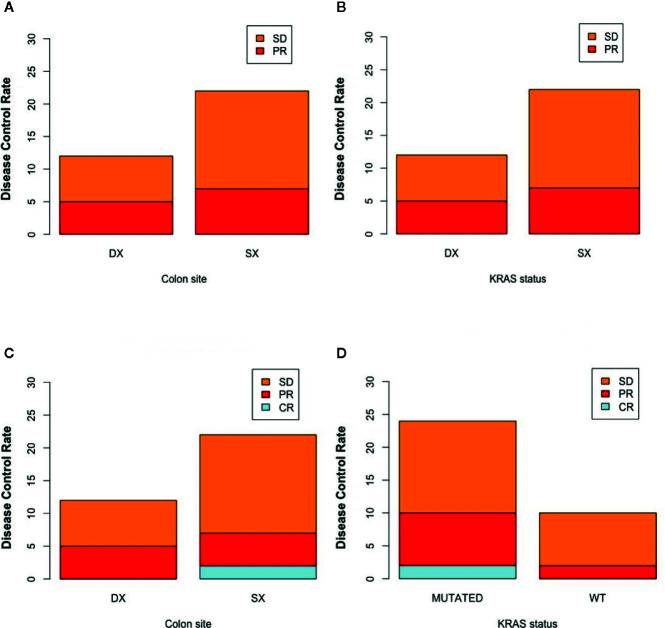
Disease control rate according to colon site and K-Ras status at timepoint-1 (respectively **A, B**) and at timepoint 2 (respectively **C, D**). SX, left colon; DX, right colon.

**Figure 2** represents the best response rate according to colon site and K-Ras status.

**Figure 3:**
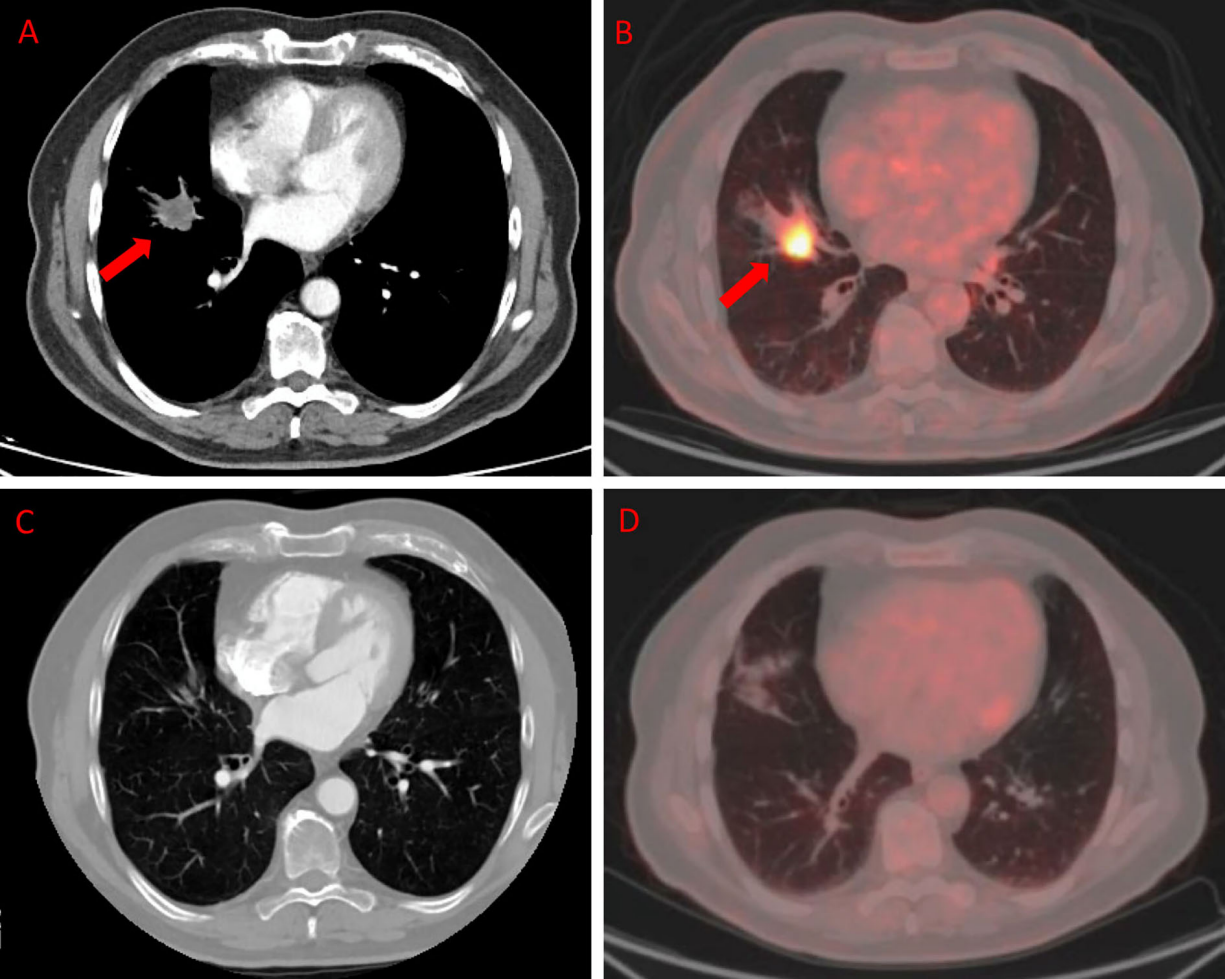
ceCT and ^18^F-FDG PET/CT in a patient affected by colon cancer with lung metastasis. A 79-year-old male affected by colorectal cancer with lung metastasis, subjected to 12 cycles of Bevacizumab-based chemotherapy and 24 DEHY sessions on the thorax as first-line therapy. Baseline ceCT **(A)** showed metastasis in the middle lobe (red arrows). Baseline whole body ^18^F-FDG PET/CT **(B)** confirmed lung involvement by the increased ^18^F-FDG uptake (red arrows) detectable axial fused PET/CT images in the same site. Timepoint-2 ceCT **(C)** and ^18^F-FDG PET/CT **(D)** evaluation demonstrated CR of lung metastasis.

**Figure 4:**
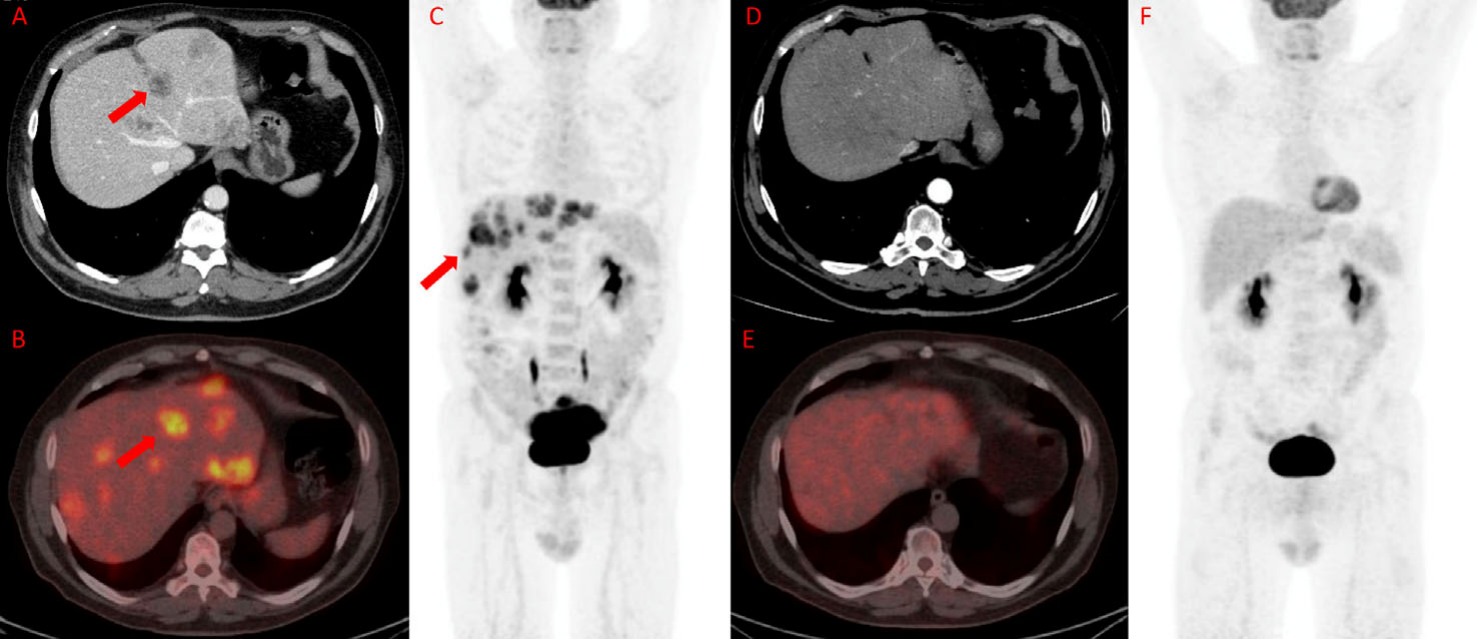
^18^F-FDG PET/TC in a patient affected by colon cancer with liver metastasis. A 55-year-old male affected by colorectal cancer with multiple liver metastases, subjected to 12 cycles of Bevacizumab-based chemotherapy and 24 hyperthermia sessions on the abdomen as first-line. Baseline ceCT showed massive liver involvement (A and red arrows) and whole-body ^18^F-FDG PET/TC showed increased ^18^F-FDG uptake in the liver lesions (red arrows) detectable also on axial fused PET/CT and MIP images **(B** and **C)** in the same site. Timepoint-2 ceCT **(D)** evaluation demonstrated significant size decrease of liver metastasis with no evidence of ^18^F-FDG uptake on whole-body PET/CT **(E** and **F)**. According to RECIST, the patient was classified as CR.

**Figures 3** and **4** represent two exemplar cases of patients judged to be in PR and CR.

**Figure 5:**
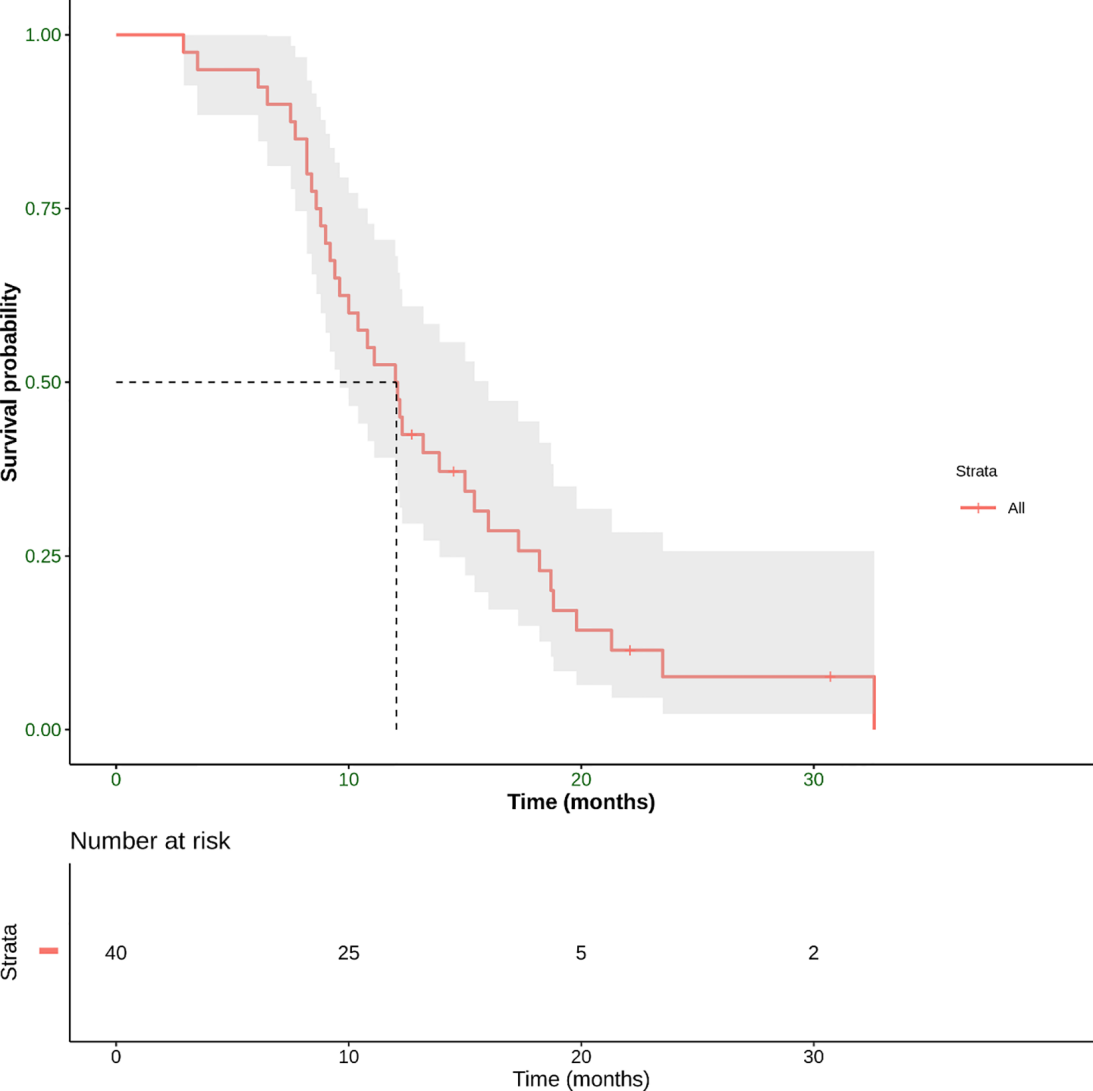
Progression-free Survival (PFS) defined as the time from the start of treatment until the date of the first radiological evidence of PD or the date of death derived from any cause, whichever occurred first. This panel shows Kaplan–Meier estimate of PFS in our patient population. The mean PFS was 12.1 months (range 3.5–32.6 months).

Median PFS, the other primary endpoint, was 12.1 months (range 2.9–32.6 months) (**Figure 5**).

**Figure 6:**
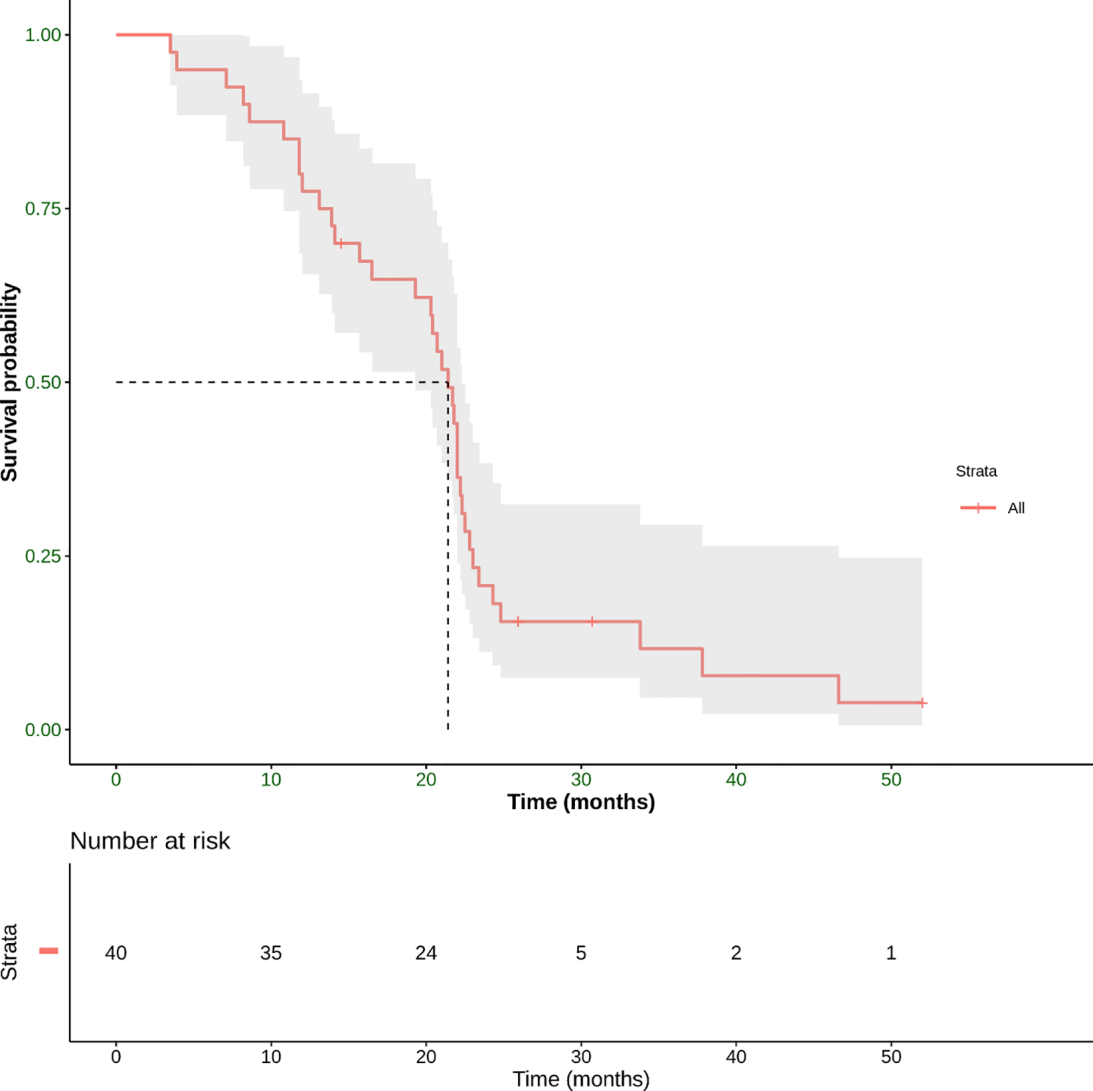
Overall survival (OS) defined as the time from the start of treatment until the date of death. This panel shows Kaplan–Meier estimate of OS in our patient population. The mean OS was 21.4 months (range 3.5–52 months).

Concerning the secondary endpoint, median OS was 21.4 months (range 3.5–52 months) (**Figure 6**).

Three patients (7.5%) underwent an attempt at curative metastasectomy obtaining complete R0 resection of the whole disease.

31 of 40 patients (77.5%) crossed over to receive a second-line therapy after disease progression. The most common regimens used were: FOLFIRI plus aflibercept (71%) or panitumumab/cetuximab (29%) based on biomarker status of the tumor. A small group also received a third-line therapy.

**Figure 7:**
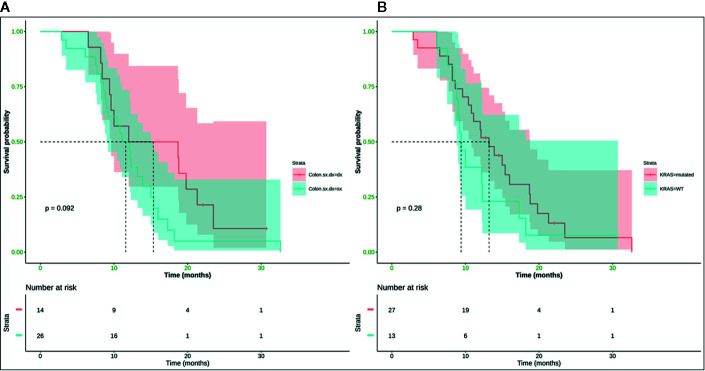
No statistically significant difference in terms of mPFS was observed (*p*-value = 0.092) between right (mPFS 15.3 months) and left (mPFS 11.6 months) colon cancer **(A)**, and (*p*-value = 0.28) between K-Ras wild type (mPFS 13.2 months) and K-Ras mutated (mPFS 9.4 months) patients **(B)**. SX, left colon; DX, right colon.

**Figure 8:**
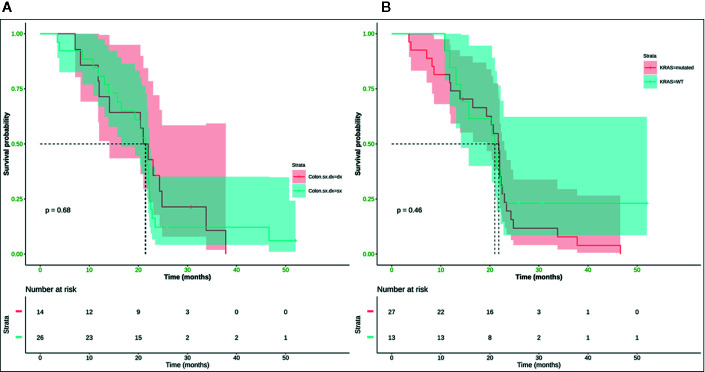
No statistically significant difference in terms of mOS was observed (*p*-value = 0.68) between right (mOS 21.5 months) and left (mOS 21.4 months) colon cancer **(A)** and (*p*-value = 0.46) between K-Ras wild type (mOS 21 months) and K-Ras mutated (mOS 21.8 months) patients **(B)**. SX, left colon; DX, right colon.

**Figure 9:**
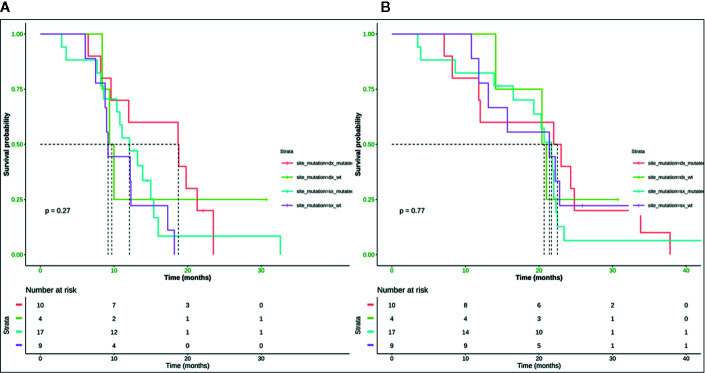
Median PFS for mutated right colon was 18.8 months, for wild type right colon was 9.7 months, for mutated left colon was 12.1 months, wild type left colon was 9.8 months **(A)**. Median OS for mutated right colon was 22.5 months, for wild type right colon was 20.7 months, for mutated left colon was 21.7 months, for wild type left colon was 21.4 month **(B)**. SX, left colon; DX, right colon.

Neither K-Ras status (p = 0.68; p = 0.48) (**Figures 7A**, **8A**) nor primitive site (p = 0.092; p = 0.68) (**Figures 7B**, **8B**), as well as any clinic-pathological variables resulted in influencing PFS and OS significantly. A Cox-hazard regression analysis has been performed aggregating K-Ras mutational status and tumor sidedness. Considering the small sample size, surprisingly, we found that patients with K-Ras wild type left-sided tumors had a double risk to have a shorter PFS [HR: 2.58 (95%CI: 0.96 ÷ 6.92)] (**Table 3** **;** **Figure 9**).

**Table 3:** Cox-hazard regression analysis results.

OS			
Variable		HR (95%CI)	p-value
KRAS mutational status and sidedness	Right colon/KRAS mutated		Ref
	Right colon/KRAS WT	0.98 (0.26 ÷ 3.62)	0.98
	Left colon/KRAS mutated	1.35 (0.59 ÷ 3.06)	0.46
	Left colon/KRAS WT	0.87 (0.32 ÷ 2.31)	0.78
**PFS**			
	Right colon/KRAS mutated		Ref
	Right colon/KRAS WT	1.23 (0.33 ÷ 4.65)	0.75
	Left colon/KRAS mutated	1.75 (0.57 ÷ 0.72)	0.21
	Left colon/KRAS WT	2.58 (0.96 ÷ 6.92)	**0.05**

Bold values denote statistical significance at the p < 0.05 level.

### Toxicity

All AEs reported in this study are shown in **Table 4**.

**Table 4:** Adverse events.

Events	No. (%)
Hematologic	
Leucopenia	6 (15%)
Anemia	5 (12.5%)
Thrombocytopenia	3 (7.5%)
Non-hematologic	
Nausea and vomiting	9 (22.5%)
Positional pain	4 (10%)
Fatigue	4 (10%)
Erythema	3 (7.5%)
Peripheralsensoryneuropathy	2 (5%)
High blood pressure	2 (5%)
Epistaxis	2 (5%)
Gastrointestinal discomfort	2 (5%)
Power-related pain	2 (5%)

Bevacizumab-FOLFOX4 regimen was substantially well-tolerated: only one patient interrupted this therapeutic scheme for sensory neuropathy, continuing treatment with bevacizumab and fluorouracil/folinic acid. In spite of this premature suspension of oxaliplatin infusions, he achieved a good response to treatment.

The main non-hematological AEs were nausea and vomiting, which occurred in nine patients. Other AEs were fatigue (four cases), peripheral sensory neuropathy (three cases), high blood pressure (two cases), epistaxis (two cases) and gastrointestinal discomfort (two cases). As for hematological AEs, leucopenia in six cases (three patients required granulocyte-colony stimulating factor), anemia (5 cases), and thrombocytopenia (three cases) were observed. However, none of the listed AEs led to a break-up of the treatment.

The addition of DEHY did not result in additional AEs on chemotherapy-related toxicity. The main DEHY-related AE was mild positional pain during treatment sessions which occurred in four patients. Erythema in the target area of DEHY was observed in three patients; power-related pain occurred in two cases during the first session and solved by power adjustment.

No patient required a significant treatment interruption because of treatment complications nor resistance phenomena to DEHY were ever observed due to the mild weekly administration, whose specific aim was to catalyze vasodilatation and a major uptake of drug in the target site. In light of our results, we can affirm that no major toxicity has been observed and reported by patients.

## Discussion

In literature, several reports demonstrate that the addition of bevacizumab to chemotherapy improves OS and/or PFS for patients affected by untreated mCRC. As reported by Kabbinavar et al., in randomized phase II studies, mCRC treated with bevacizumab and 5-FU/LV obtained an OS improvement with respect to the same regimen without bevacizumab (8, 10, 11). Hurwitz H. et al. conducted a phase III trial comparing irinotecan, bolus fluorouracil, and leucovorin (IFL) with and without bevacizumab demonstrating an increased mOS in the bevacizumab group (9). Moreover, the combination of bevacizumab with FOLFOX-4 or XELOX resulted in a statistically significant improvement in mPFS compared with those patients treated with chemotherapy alone (hazard ratio [HR], 0.83; 97.5% CI, 0.72 to 0.95; *p* = .0023), while no statistically significant difference in mOS was reached (HR, 0.89; 97.5% CI, 0.76 to 1.03; *p* = .077) (NO16966 trial) (12). The last reported phase III trial is one of the registered clinical studies of bevacizumab in its first commercial formulation Avastin^®^, which contributed to the final approval of the drug for therapy. To our aims, the critical end-point results of this trial have represented the right and immediate comparison group, lacking in our study an internal control patient group randomized to the same chemotherapy treatment without DEHY.

Bevacizumab binds VEGF, the key promoter of vasculogenesis and angiogenesis, preventing its bond to the specific receptors, Flt-1 (VEGFR-1) and KDR (VEGFR-2), on the surface of endothelial cells. Therefore, bevacizumab blocks the biological activity of VEGF, reverses the vascularization of tumors, normalizes the residual tumor vascularization and inhibits the formation of new vascularization, thus preventing tumor growth (80).

Its complex tridimensional structure has a molecular weight of 149 kDa; its bioavailability is 100% only by intravenous administration, and its half-life of about 20 days (range: 11–50 days) is compatible with the frequency of standard chemotherapy in mCRC, as well as for other chemotherapeutic schedules in different tumors. This favorable half-life is due to its peculiar metabolic and elimination profiles which are comparable to native IgGs, unable to link VEGF. It is initially attacked by proteolytic enzymes everywhere in the body, including endothelial cells and is not principally eliminated through liver or kidney because IgG link with FcRn receptor protects them from elimination, conferring a long terminal half-life. Bevacizumab’s clearance value of 0.231 l/die completes its pharmacokinetic profile, summarizing that initial half-life of 1.4 days and a terminal half-life of 20 days are comparable to native IgG terminal half-life, swinging from 18 to 23 days.

In this contest, the combination therapy bevacizumab plus DEHY finds a fertile field of application in clinics.

More recent anti-tumoral combination strategies have proven to be helpful to bevacizumab increasing its activity and clinical efficacy. In particular, HIF-1 inhibitors having limited activity as single agents, proved to enhance bevacizumab efficacy by contrasting the intratumor-hypoxia it induces by increasing HIF-1 dependent gene in the target tissue. Experimental models testing bevacizumab in combination with the HIF-1 inhibitor topotecan proved that this association generates a profound inhibition of HIF-1 transcriptional activity, a more significant inhibition of proliferation than bevacizumab alone, and the induction of apoptosis that bevacizumab alone is unable to induce. Waiting for further results about this pharmacologic combination strategy, the inhibition of HIF-1 expression can be physically reached, thanks to hyperthermia.

HT has an anti-cancer effect by means of multiple direct and indirect mechanisms including the inhibition of angiogenesis. HT can explicate this action through the direct damage of endothelial cells, the vasodilatation that enhances tumor reoxygenation, and by decreasing oxygen consumption (70, 72, 73). These mechanisms reduce tumor hypoxia and, subsequently, the expression of HIF-1 which plays a central role in the regulation of angiogenesis and cell metabolism. Specifically, HIF-1 is a transcription factor that triggers VEGF expression, the most powerful angiogenic factor, and shifts cells towards glycolysis decreasing oxygen consumption rate. By combining the two agents, bevacizumab and HT, the synergism of action, the goal they share and the possibility to organize subsequent administrations with an additional intermediate cycle of HT can probably further lengthen bevacizumab’s terminal half-life so that a major and safe accumulation of drug due to HT vasodilatation can occur in the target district. Moreover, HT is able to contrast HIF-1 gene expression induced by bevacizumab, leading to a stable negative balance of this tumor marker.

These are perhaps hypothesis to explain the longer mPFS than historical control (NO16966 trial) in our population and could also predict even better data of mOS than standard treatment still to prove.

To the best of our knowledge, no authors investigated the possible clinical advantages deriving from the combination of bevacizumab plus FOLFOX-4 with DEHY in untreated mCC. In a previous pilot study, we evaluated bevacizumab–chemotherapy combined with DEHY in multi-treated patients affected by colorectal, breast, and ovarian cancers.

Our data demonstrated that DEHY may enhance the bevacizumab-based treatment, in particular improving tumor response (78).

Based on the possible biological and clinical interaction between bevacizumab and DEHY effects, we conducted a pilot study to analyze the efficacy of bevacizumab plus FOLFOX-4 combined with DEHY in untreated mCC patients.

Our results showed that this combination determined a high disease control. In more detail, we obtained a DCR of 95 and 89.5% at timepoint-1 and timepoint-2, respectively. The median value of PFS was 12.1 months, while median OS was 21.4 months. The other important result was the absence of major toxicity related to DEHY.

Another result of our statistical Cox-analysis was the discovery that patients with KRAS wild type left-sided tumors have a double risk to have a shorter PFS.

Therefore, our study suggests that the addition of DEHY to bevacizumab-FOLFOX-4 regimen enhances the efficacy of the gold standard treatment, as studied in NO16966 clinical trial, through a high disease control and a longer mPFS (our report: 12.1 months *vs* historical control: 9.4 months) without additional adverse events or chemotherapy-related toxicity. The second clinical parameter we evaluated, DCR, cannot be directly compared to the response rate (RR) in NO16966, where there was no specific reference to times within the course of clinical evaluations. We chose to study DCR as a natural continuation of our previous pilot study, where we observed through DCR very promising stabilized diseases and objective responses.

Although NO16966 represents a historical comparison, our results support the validity of this study since the baseline patient characteristics of enrolled population, the inclusion and exclusion criteria, and the administered chemotherapy regimen are comparable to the historical control.

Moreover, the main limitations of our study, the small sample size, and the absence of comparison with an internal control group, did not allow us to adequately analyze mOS that, in relation to mPFS data, could prove to be longer than the standard treatment.

In spite of these aspects, we succeeded in focusing on a homogeneous patient population, and our results represent the first clinical data on the potential benefit of DEHY in addition to bevacizumab-based chemotherapy. We also demonstrated the safety of this type of combination treatment due to a different specific use of DEHY as catalyzing agent of vasodilatation and drug uptake.

## Conclusion

Bevacizumab plus FOLFOX-4 combined with DEHY as first-line therapy in colon cancer patients demonstrated both tolerability and efficacy. Bevacizumab’s pharmacokinetic data will interweave with DEHY’s ability to retain drugs in the selected treatment areas so that drug elimination undergoes a delay compatible with our results of the principal clinical endpoints. Moreover, their opposite effects on HIF-1 expression play a key role in controlling disease progression and represent a new field of research in oncology. This regimen, as adopted in our study, has also demonstrated that DEHY in combination with chemotherapy schedules has no relevant toxicity, is safe and receives acceptable compliance by patients. A randomized trial will be necessary in the near future to further confirm these data.

## Data Availability Statement

The raw data supporting the conclusions of this article will be made available by the authors, without undue reservation.

## Ethics Statement

Hyperthermia is recognized and reimbursed by Italian Health System therapeutic strategy in association with chemotherapy or radiotherapy in the treatment of tumors, being identified by the International Classification of Diseases, Ninth Revision, Clinical Modification (ICD-9-CM) with the code 9985. Consequently, this treatment does not need a clinical trial, but only the signed informed consent. The patients/participants provided their written informed consent to participate in this study.

## Author Contributions

Conceptualization, GRa, CL, and CG. Methodology, SDS. Software, SDS, MP. Validation, FM. Formal analysis, MA. Investigation, PM. Resources, GL, CG. Writing—original draft preparation, CL, ML. Writing—review and editing, ML. Visualization, AP, GRu, CF. Supervision, GRa, CG. Project administration, GRa, CL. All authors contributed to the article and approved the submitted version.

## Conflict of Interest

The authors have no relevant affiliation or financial involvement with any organization or entity with a financial interest in or financial conflict with the subject matter or materials discussed in the manuscript.
