# Corrigendum: Bevacizumab Plus FOLFOX-4 Combined With Deep Electro-Hyperthermia as First-line Therapy in Metastatic Colon Cancer: A Pilot Study

**DOI:** 10.3389/fonc.2020.637880

**Published:** 2021-02-05

**Authors:** Girolamo Ranieri, Carmelo Laface, Mariarita Laforgia, Simona De Summa, Mariangela Porcelli, Francesco Macina, Michele Ammendola, Pasquale Molinari, Gianfranco Lauletta, Alessandra Di Palo, Giuseppe Rubini, Cristina Ferrari, Cosmo Damiano Gadaleta

**Affiliations:** ^1^ Interventional and Medical Oncology Unit, IRCCS Istituto Tumori “G. Paolo II”, Bari, Italy; ^2^ Pharmacy Unit, IRCCS Istituto Tumori “G. Paolo II”, Bari, Italy; ^3^ Molecular Diagnostics and Pharmacogenetics Unit, IRCCS Istituto Tumori “Giovanni Paolo II”, Bari, Italy; ^4^ Department of Health Science, Digestive Surgery Unit, University “Magna Graecia” Medical School, Germaneto, Italy; ^5^ Department of Biomedical Sciences and Human Oncology, Section of Internal Medicine “G. Baccelli”, University of Bari Medical School, Bari, Italy; ^6^ Nuclear Medicine Unit, D.I.M., University of Bari “Aldo Moro”, Bari, Italy

**Keywords:** tumor angiogenesis, bevacizumab, hyperthermia, chemotherapy, metastatic colon cancer

In the original article, the ethical consideration statement was inserted by error in the section **Patients and Methods**, sub-section **Statistical Analysis**, as paragraphs 3 and 4:

“The study was performed in accordance with the Declaration of Helsinki and Good Clinical Practice Guidelines. The Internal Ethical Committee approved the study without reserve together with the module for patients’ informed consent (Trial n° 871 of the internal protocol numbering).

Written informed consent was obtained from all patients before study participation.”

The corrected **Statistical Analysis** section with the paragraphs removed appears below:

“The primary endpoints were disease control rate (DCR) and PFS. The secondary endpoint was OS. DCR was considered as the percentage of patients who had the best response rating [complete response (CR), partial response (PR), or stable disease (SD)] and was assessed at 90 days (timepoint-1) and at 180 days (timepoint-2).

PFS was defined as the time from the start of treatment until the date of the first radiological evidence of PD or the date of death derived from any cause, whichever occurred first. OS was specified as the time from the start of treatment until the date of death.

Fisher’s exact test was used to assess the correlation between DCR, PFS, OS, and tumor location (left-sided CRC/right-sided CRC), K-RAS status (wild type/mutation), number of metastatic sites (1–2, ≥3), liver involvement (yes/no), and/or lung involvement (yes/no). R barplot() function was used to create barplots. For survival analyses, the Kaplan–Meier method was used to estimate the correlation between PFS, OS rates, and clinic-pathological variables at 95% CI. The log-rank test was used to compare survival curves. The “survival” R package has been used to perform survival analyses. Cox proportional hazards regression test using the ‘coxph’ function of the R ‘survival’ package has been elaborated. Survival curves have been graphically depicted by “ggplot 2” R package. All statistical analyses were performed using R version 3.6.”

The **Ethics Statement** section was misleading as it appeared in the published version:

“The studies involving human participants were reviewed and approved by Comitato Etico Istituto Tumori “Giovanni Paolo II” Istituto di Ricovero a carattere scientifico. The patients/participants provided their written informed consent to participate in this study.”

The corrected **Ethics Statement** appears below:

“Hyperthermia is recognized and reimbursed by Italian Health System therapeutic strategy in association with chemotherapy or radiotherapy in the treatment of tumors, being identified by the International Classification of Diseases, Ninth Revision, Clinical Modification (ICD-9-CM) with the code 9985. Consequently, this treatment does not need a clinical trial, but only the signed informed consent. The patients/participants provided their written informed consent to participate in this study.”

The authors apologize for this error and state that this does not change the scientific conclusions of the article in any way. The original article has been updated.

